# Levels and Determinants of Health Insurance Coverage in Kenya: Cross-Sectional Evidence from KDHS 2022

**DOI:** 10.3390/healthcare14121648

**Published:** 2026-06-10

**Authors:** Maha Alhajeri, Elham Aldousari, Dennis Kithinji

**Affiliations:** 1 Department of Health Informatics and Information Management, College of Allied Health Sciences, Kuwait University, Sulaibikhat 90805, Kuwait; elham.aldousari@ku.edu.kw; 2 Department of Medical Laboratory Sciences, College of Health Sciences, Meru University of Science and Technology, Meru 60200, Kenya; 3 Department of Research and Writing, Medright Consulting Ltd., Maua 60600, Kenya

**Keywords:** health insurance, Kenya, Social Health Authority, media, National Hospital Insurance Fund, Social Health Insurance Fund, universal health coverage, wealth, education level

## Abstract

**Highlights:**

**What are the main findings?**

- According to the 2022 Kenya Demographic and Health Survey (KDHS 2022), only about a third of KDHS 2022 participants had health insurance coverage just before the transition from the National Hospital Insurance Fund (NHIF) to the Social Health Authority (SHA).
- Education level, wealth index, ownership of media devices, and recent outpatient care significantly predicted the health insurance coverage status of KDHS 2022 participants.

**What are the implications of the main findings?**

- Reforms envisaged in the transition from the NHIF to SHA were necessary to increase health insurance coverage among Kenyans.
- The SHA will successfully finance healthcare access for all Kenyans if its funding is aligned with education levels, economic status, and access to media devices among Kenyans.

**Abstract:**

**Background/Objectives**: Strategies to improve the Social Health Authority (SHA)’s equity can be identified by analyzing the Kenya Demographic and Health Survey (KDHS) 2022. This study reports evidence of determinants of health insurance coverage in Kenya. **Methods**: Household- and individual-level datasets from the Kenya Demographic and Health Survey conducted between February and July 2022 were combined to form the analyzed dataset. Proportions of individuals with and without health insurance were estimated. The associations between potential determinants and health insurance status were calculated using the Rao–Scott chi-square. Logistic regression was used to analyze the determinants of health insurance coverage. **Results**: Most of the 14,232 participants were literate (75%), relatively poor (56%), in good health (79%), connected to electricity (55%), and radio listeners (61%). About 34% had health insurance, with 93% of the insured covered by the NHIF. Twenty predictors (Adjusted F = 4.2–434.1, *p* < 0.0001) were included in the complex sample logistic regression model, but only nine were statistically significant predictors of health insurance coverage. The key predictors were education level; wealth index; ownership of a solar panel, television, smartphone, and computer; age; and recent outpatient care (11–80% differences in odds). **Conclusions**: Health insurance coverage remains low in Kenya due to low education levels, poor economic status, and disparities in access to media. The SHA can emphasize media campaigns in the informal sector to increase premium payments. Accelerating socioeconomic advancement and adopting tax-based funding could speed up Kenya’s progress towards UHC.

## 1. Introduction

Kenya leveraged social health insurance through the National Hospital Insurance Fund (NHIF) from 1966 to 2024 and transitioned to the Social Health Authority (SHA) from October 2024 onwards as its principal pathway to universal health coverage (UHC) [1]. Kenyans mandatorily contribute to the Social Health Insurance Fund (SHIF) to finance Kenya’s journey to UHC, which was also the case for the NHIF [2]. Kenyans in the informal sector barely contributed to the NHIF and now the SHIF since the government lacks a mechanism to automatically deduct the premiums from their income, as is the case in the formal sector [3]. Kenya seeks to leverage the SHA to finance healthcare through the premium-based SHIF and tax-funded and donor-funded Primary Healthcare Fund (PHF) and Emergency, Chronic, and Critical Illness Fund (ECCIF) [1]. Accordingly, the SHA mainly relies on a premium-based model of financing social health insurance supplemented by tax-funded components.

In contrast, Rwanda uses community-based health insurance (CBHI), which is substantially donor-funded but backed by few ($3.2 per member annually) wealth category-based premium contributions and multiple government revenue sources to achieve high insurance levels [4]. Rwanda’s donor dependence is unsustainable and may not work in Kenya since Kenya’s population is four times larger; thus, its health insurance cannot be sustained by donor funding. Low- and middle-income countries have unique health system challenges; hence, each country requires targeted health financing interventions [5]. In Kenya, the majority of the regular contributors to the SHIF are formal employees, since employers are mandated to submit their contributions to the SHA [6], and Rwanda’s model of low premiums may not be sustainable in Kenya since even Rwanda is exploring a raise in premiums for economic viability [4].

Most Kenyans in the informal sector rely on out-of-pocket payments to access healthcare services [7]. Yet the majority cannot afford quality healthcare services, since 36% of Kenyans live below the $2.15/day poverty line [8]. Kenya and Tanzania are attempting to mandate contributions to social health insurance in the informal sector, but enforcing such legislation is difficult due to unaffordability and general-public perceptions of low value for money [5]. Similarly, the willingness to pay premiums for community-based health insurance (CBHI) in Uganda is 0.2%, which is the overall average in Africa [9]. Therefore, access to healthcare services is limited for most Kenyans and other people in Africa at large.

The need for functional and equitable social health insurance in Kenya increased in 2025 due to the budget cuts for USAID and the World Health Organization [10], which have been supporting several health programs in Kenya. The US government paused aid, which led to the discontinuation of PEPFAR in January 2025, and five countries that fund 90% of HIV care globally announced aid reductions ranging from 8 to 70% [10]. The SHA’s approach—to combine premium contributions mainly from the formal sector with tax funding—can steer progress towards higher health insurance coverage levels, as evidenced in Ghana [11]. However, Kenya continues to mainly rely on premium contributions from the informal sector [12], and the challenge of low rates of premium contributions that affected the NHIF persists in the SHA.

Health insurance uptake in Kenya has been low, with only about 20% having health insurance coverage based on the 2014 Kenya Demographic and Health Survey (KDHS) [13]. Low health insurance coverage is a common phenomenon in sub-Saharan Africa—a 2021 study that analyzed the most recent DHS data in 36 countries in sub-Saharan Africa found only four countries having health insurance coverage levels above 20%: Rwanda (79%), Ghana (58%), Gabon (41%), and Burundi (22%) [14]. Kenya’s health insurance coverage level was below the 27% average for lower-middle-income countries (LMICs) [15]. Therefore, Kenya is underperforming in terms of increasing its health insurance coverage levels, since even recent increases in SHA registrations are barely accompanied by consistent SHIF contributions.

Health insurance coverage is a demographic and socioeconomic issue in LMICs [4]; hence, it is best studied in the context of underlying determinants to identify avenues for targeted equity-oriented interventions. Kenya’s social health insurance is the backbone of financial protection towards UHC, yet the main beneficiaries are active SHIF members, who are mainly in the formal sector, which only employs about 23% of Kenyan workers [16]. The government of Kenya intervenes through mass campaigns to convince Kenyans in the informal sector to regularly contribute to social health insurance, but the compliance rates remain low [17]. Identifying determinants of health insurance coverage can further guide the government and other stakeholders in designing targeted interventions to increase the number of active SHIF members.

In the 2014 KDHS, older age, male gender, marriage, a small or rich household, chronic disease, and exposure to media were associated with health insurance coverage [13]. Media exposure and digital health technologies, which have been consistently growing in Kenya, facilitate the enrolment and maintenance of populations in health insurance schemes in LMICs [18,19]. Another KDHS was conducted in 2022; hence, analyzing the data to identify more specific and modifiable factors behind health insurance coverage, particularly regarding media and digital access, is necessary. This can inform the design of interventions to increase individual contributions to the recently launched SHIF.

The KDHS 2022 reported that only about 20% of Kenyans aged 15–54 years had more than secondary education, although over 90% were literate based on either having a post-secondary education or the ability to read a sentence presented to them [20]. Radio and television were the commonest means of media exposure, with more than 60% of the participants either listening to the radio or watching television. More than half of the participants used the internet on a daily basis [20]. Therefore, education levels, listening to the radio, watching television, and browsing the internet may be influencing their health insurance coverage.

As mentioned, according to the KDHS, only 26% of Kenyans aged 15–54 years had health insurance in 2022, with the majority being urban dwellers (40%) compared to rural residents (19%). Households in urban areas had higher rates of health insurance coverage (46% in Nairobi City County) compared to households in rural areas (5% in Tana River County) [20]. Cash payment was the main approach to health expenditures (KSh 13,621 per year per household), followed by NHIF payments (KSh 9330 per year per household), with urban dwellers making approximately twice as many cash payments as rural residents for health services [20].

The inequitable distribution of health insurance coverage and spending on health calls for the identification of modifiable predisposing factors that can be targeted to reduce inequities in health access for progress towards UHC across Kenya [21]. However, the most recent publication on the determinants of health insurance in Kenya that we could retrieve from a literature search in May 2025 analyzed the KDHS 2014 dataset, yet the KDHS 2022 is available [13]. The KDHS 2022 was conducted after NHIF expansion in 2015 [22], the piloting of UHC in four counties in 2018 [23], and the COVID-19 pandemic between 2020 and 2023 [24]; thus, substantial changes may have occurred in both health insurance coverage and its determinants.

The KDHS 2022 has more variables regarding health insurance coverage, health expenditure, socioeconomic context, and media exposure compared to the KDHS 2014 [20], making it a richer data source for the identification of specific determinants of health insurance coverage. For example, in addition to data on having any health insurance coverage, as in the KDHS 2014, the KDHS 2022 specifies the types of health insurance coverage [20]. It also allows the linkage of health insurance coverage data to employment status, education levels, household characteristics, and the wealth index compared to the KDHS 2014 [20]. Hence, analyzing the KDHS 2022 dataset is likely to reveal more precise and policy-relevant determinants of health insurance coverage. This study aimed to identify predictors of health insurance coverage among Kenyans aged 15–54 years by analyzing the KDHS 2022 dataset.

## 2. Materials and Methods

### 2.1. Setting

Kenya, a developing country that uses a social health insurance model to advance towards UHC, was the context for this study. Kenya transitioned from the NHIF to the SHA in October 2024 to streamline financing and operational efficiency. As of February 2025, the SHA had almost 20 million registrations, but active SHIF contributors amounted to only 3.5 million people, mainly the mandatorily contributing formal employees [25]. The Kenyan government is committed to the registration of all Kenyans with the SHA and the full operationalization of the SHA [26]. It allocates government revenue to the PHF and ECCIF, as well as the SHIF for indigent households identified using the means testing instrument [12]. The KDHS 2022, which contains health insurance coverage data, is the 7th and the most recent KDHS as of May 2025, when this study was conducted. It collected data from 17 February 2022 to 31 July 2022 [20].

### 2.2. Study Design

A cross-sectional study design was implemented using the Kenya Demographic and Health Survey (KDHS) 2022 data to estimate the level of insurance coverage among Kenyans aged 15–54 years and identify predictors of health insurance coverage in Kenya. The study is reported in accordance with the STROBE recommendations where applicable [27].

### 2.3. Participants

Data from individuals and households where KDHS 2022 data were collected across Kenya were included in the study. Variables regarding health insurance coverage and potential determinants of health insurance coverage were included in the analyzed dataset.

The KDHS 2022 applied a two-stage stratified sampling design with stratification for rural and urban residence. Its sampling frame was the 2019 Population and Housing Census. Probability proportional to size was applied to select enumeration areas (clusters/primary sampling units). Then, 25–30 households were selected, equal to the probability, from each cluster after listing households to form the Household Recode (HR) dataset. Women aged 15–49 in the selected households, who had spent the night before the survey in the household or were the usual residents of the household, were successfully interviewed to form the Individual Recode dataset. Men aged 15–54 years, who were usual residents or visitors who had spent the night before the survey in half of the households selected for the Individual Recode (IR) dataset, were interviewed to create the Men Recode (MR) dataset.

The sample in the KDHS 2022 included 1691 clusters across Kenya—666 in urban areas and 1025 in rural areas, selected using the equal probability selection method (EPSEM). Twenty-five households were selected per cluster; a total of 42,022 households were included, since some clusters had fewer than 25 households.

### 2.4. Variables

The dependent variable was the health insurance coverage of the individuals. The independent variables were education attainment, literacy, the wealth index, indicators of access to media (having electricity, solar panels, a radio, a television, a telephone, a mobile phone, a computer, and internet access and reading newspapers), health status, receiving outpatient and inpatient care, paying for care, the median outpatient and inpatient cost, gender, and age [20].

### 2.5. Data Source—Retrieval of KDHS 2022 Data

The KDHS 2022 data were obtained from the Kenya National Data Archive (KeNADA) [28]. Data on health expenditure and health insurance coverage in the KDHS 2022 were collected from 21,996 households. The HR (household characteristics), IR (women aged 15–49 years), and MR (men aged 15–54 years) datasets were combined to form the dataset that was analyzed in this study. The KDHS created these three datasets from data collected through computer-assisted personal interviews in preselected households. The IR and MR datasets comprised demographic data like age, socioeconomic data such as the highest level of education, and health-related data including health insurance coverage for women and men, which were not included in the HR dataset.

The household characteristics of interest in the HR dataset were socioeconomic and structural characteristics that influenced health insurance coverage but were not included in the individual-level datasets. The three datasets had common identifiers (cluster number, household number, and respondent’s line number), which facilitated the merging of the datasets without duplication, inconsistencies, or artificial missing data. Adding variables in the HR dataset to the merged IR and MR datasets as explanatory variables prevented the introduction of missing values during merging.

### 2.6. Bias

Sampling in the KDHS 2022 was performed without replacement so as to avoid duplication, which would have reduced the representativeness of the sample and introduced bias in the estimation of population parameters.

### 2.7. Ethical Considerations

This study analyzed an anonymized Public Use Dataset from the KDHS, collected and maintained in line with strict ethical guidelines by the Kenya National Bureau of Statistics (KNBS), in collaboration with international partners. The KNBS obtained ethical approval from the Kenya Medical Research Institute (KEMRI) Scientific and Ethics Review Unit, while the ICF Institutional Review Board reviewed the protocols to ensure ethical compliance. No further ethical clearance was needed because the study was a secondary analysis of publicly available KDHS data. A request to access the KDHS 2022 data for a research study was submitted to the KNBS and granted. All access conditions of the KNBS were observed. The authors did not have access to information that could identify individual participants during or after data collection.

### 2.8. Data Analysis

Data analysis was conducted using IBM SPSS Statistics for Windows, Version 25.0 (IBM Corp., Armonk, NY, USA). List-wise deletion was applied to manage missing data. The analyses were focused on associations between variables for hypothesis testing, rather than obtaining nationally representative estimates. Nevertheless, the analyses accounted for weighting, clustering, and stratification in the complex sampling design of the KDHS 2022. Frequencies of health insurance coverage and its potential determinants were obtained in counts and percentages. The complex samples crosstabs procedure was used in the tests of independence to generate Rao–Scott chi-square values. The adjusted chi-square was converted to a design-based F-statistic to estimate the associations between the potential determinants and status of health insurance coverage. Taylor-series linearization was used to estimate the associated numerator (df1) and denominator (df2) degrees of freedom and the standard errors.

All significant factors (*p* < 0.05) in the complex samples crosstabs were included in a complex samples multivariable logistic regression model. This is because the outcome was binary (having or lacking health insurance coverage), and the aim was to identify determinants of health insurance coverage. Including the stratification variable in the sampling plan resulted in several strata with degenerate insurance status data, which prevented model estimation. Thus, the final complex samples plan only included the weighting and cluster variables, which allowed the adjustment of the model for the survey design during convergence to prevent standard errors in clustering. The KDHS 2022 weights were normalized before analysis to prevent the inflation of the sample size during the logistic regression. Weighted maximum likelihood was used to estimate coefficients for validity in the estimation of odds ratios, confidence intervals, and *p*-values. A sensitivity analysis was not conducted since the study used standardized variables, specified the model based on the prior literature, had minimal missing data, did not use complete-case analysis, and accounted for the complex survey design in all analyses.

## 3. Results

### 3.1. Descriptive Results: Characteristics of the Participants

Out of the 14,232 respondents aged 15–54 years old whose status of health insurance coverage were indicated in the KDHS 2022 dataset, 33.9% were covered by health insurance (Table 1). Their mean age was 32.9 years (standard deviation, SD = 9.1), with 65% being 26–45 year olds.

The total outpatient costs varied significantly, from as low as zero (*n* = 58) to as high as KSh 530,000 (*n* = 1). Only 3.1% (*n* = 84) of the outpatient costs exceeded KSh 10,000. Cash and the NHIF were the main approaches to fulfilling outpatient healthcare costs, as 91% of the 2698 respondents paid some cash (KSh 1–130,000) for healthcare costs, with 56 of these cash payments being KSh 10,000 or more. The NHIF paid bills for 5.6% (*n* = 148) of respondents (range: KSh 100–400,000); only 18 were KSh 10,000 or more. Eight respondents indicated that the amount was met in kind (range KSh 500–20,000). Fifty-two respondents had their payments covered by private insurance (range KSh 20–100,000), with only 10 incurring payments exceeding KSh 10,000. The number of people that incurred a specific outpatient cost always exceeded the number of people whose hospital costs of a similar amount were covered by the NHIF.

The total inpatient costs varied from zero Kenyan shillings (*n* = 62) to KSh 4.2 million (*n* = 1), with 41.8% (*n* = 319) bills exceeding KSh 10,000. Sixty-two of the 763 inpatient costs were covered for free. A total of 575 cash payments for inpatient care were made, ranging from KSh 50 to KSh 950,000. The NHIF only contributed to the payment of 170 of the inpatient bills, with amounts ranging from KSh 1000 to KSh 400,000. Private insurance only contributed to paying 29 of the inpatient bills, with amounts ranging from KSh 2000 to KSh 700,000. In-kind payments ranging from KSh 560 to KSh 700,000 were made for 17 inpatient bills. Thus, most patients paid for both outpatient and inpatient care out of pocket, with some patients combining multiple funding sources to pay their inpatient bills.

### 3.2. Inferential Results: Factors Associated with Health Insurance Coverage

All factors that were included in the bivariate analysis, except gender and having paid money for the last outpatient care, were statistically significantly associated with health insurance coverage (Table 2).

### 3.3. Determinants of Health Insurance Coverage

The distribution of health insurance coverage (Yes or No) in relation to several independent variables was predicted using complex samples multivariable logistic regression. Variance inflation factors (VIFs) and tolerance were examined to assess multicollinearity. The VIF values ranged from 1.0 to 1.1; hence, multicollinearity was not a concern. Tolerance values ranged from 0.91 to 1.0; thus, the levels of collinearity among the predictors were acceptable.

Complex samples multivariable logistic regression was conducted using the forward Wald method since there were multiple potential predictors. All included predictors were categorical. The model converged in five iterations, indicating the stability of the obtained maximum likelihood estimates. The model stopped adding predictors in Step 11, in which the Omnibus Test of Model Coefficients was statistically significant, χ^2^ (23) = 3510.4, *p* < 0.0001). From Step 0 to Step 11, the −2 log likelihood decreased from 13,848.5 to 12,711.2, indicating a better fit with the added predictors. The model explained about 24.8% and 33.9% of the variance in insurance coverage status according to the Cox and Snell R^2^ and Nagelkerke R^2^, respectively.

In the final model (Step 11), nine variables were retained and all of them were significant predictors of health insurance coverage status (Table 3). Most estimates of odds ratios were precise considering the overall narrow confidence intervals. All associations except partial reading ability, the 36–45 age group, and not owning a solar panel among people in homesteads without an electricity connection were strongly negative, since the odds ratios were significantly below one and the *p*-values were equal to or less than 0.001. Inequity factors like a lack of education, poverty, and young age were the most prominent predictors of lacking health insurance coverage (65–80% lower odds). Media tools were the second most significant category of predictors, since people without them had 20–39% lower odds of having health insurance coverage.

Specifically, respondents with no education had 80% lower odds of having health insurance coverage than individuals with higher education. The poorest respondents had 73% lower odds of having health insurance coverage compared to the richest participants. Participants aged 19–25 years had 65% lower odds of having health insurance coverage compared to those aged 46–55 years. Similarly, individuals in households without a television had 39% lower odds of having health insurance coverage compared to those whose households had a television. Lacking a computer or smartphone was also associated with 27% and 20% lower odds of having health insurance coverage, respectively. People who had not received outpatient care in the last four weeks had 15% lower odds of having health insurance coverage compared to those who had received it. Among individuals in households without an electricity connection, lacking a solar panel in the household was associated with 11% lower odds of having health insurance coverage compared to having a solar panel.

## 4. Discussion

This study identified predictors of health insurance coverage among Kenyans aged 15–54 years and estimated the probability of coverage with the presence of the identified predictors by analyzing the KDHS 2022 dataset. Only about a third of Kenyans aged 15–54 years who answered the question on health insurance coverage in the KDHS 2022 were insured. Complex samples logistic regression revealed that inequity factors like lacking education, poverty, and a young age were the main predictors of lacking health insurance coverage. Media ownership factors such as lacking a TV, smartphone, computer, and source of electric power modestly predicted a lack of health insurance coverage. Lastly, having not received outpatient care in the last four weeks weakly but significantly predicted a lack of health insurance coverage. The identified determinants reveal inequity-based systemic health insurance coverage gaps, which can inform equity-oriented policy development and implementation towards financial risk protection in healthcare, as envisioned in the UHC and Sustainable Development Goal (SDG) 3.8.

The level of health insurance coverage is low in Kenya based on our findings, which align with the estimates in the KDHS 2022 report. Most Kenyans in the informal sector are uninsured [29], which explains the low coverage. Although low coverage is reported in other LMICs, countries such as Rwanda, Ghana, and Gabon have high levels of insurance coverage yet they are also in sub-Saharan Africa [15]. High enrolment rates do not translate to health insurance coverage, because more than 70% of the few NHIF members did not renew their NHIF membership [29], which explains the small number of healthcare bills paid by the NHIF while Kenyans incurred many healthcare bills.

In contrast, Rwanda’s CBHI covers more than 90% of the population and it has high rates of public compliance with the payment of premiums [4]. The difference between Kenya and Rwanda’s compliance rates could be due to the high levels of annual premiums in Kenya, since the average Kenyan pays $60, which is 20 times more than what an average Rwandese pays ($3) [4,12]. Until the majority of Kenyans in the informal sector can afford and see the value for money in SHIF contributions, they are likely to remain non-compliant and thus uncovered, since their expected contributions are relatively high. Alternatively, the SHA regulations can be revised to decrease the premiums for people in the informal sector by leveraging tax funding to compensate for the SHA’s budgetary deficit to ensure better affordability and compliance.

Most healthcare costs were paid out of pocket as the NHIF sometimes paid nothing or only paid a small fraction of both the outpatient and inpatient bills. In contrast, less than 10% of healthcare expenditures in Thailand are paid out of pocket [30]. The NHIF’s low purchasing power, despite the 2015 reforms that sought to leverage it to steer Kenya towards UHC, could be the reason for the discrepancies between the healthcare costs and NHIF payments [31]. Additionally, the premium contributions and the benefits package do not appeal to people in the informal sector; hence, they choose to remain uninsured [31]. Therefore, the SHA should implement stricter quality assurance measures so that people in the informal sector can access SHA-paid comprehensive quality care for early demonstrable value for money.

Moreover, the diversity of benefits packages among contributors – the informal sector lacking access to pertinent services compared to civil servants has discouraged Kenyans in the informal sector from contributing to the NHIF [29]. In contrast, Thailand’s UHC system ensures access to care and the financial protection of its beneficiaries, even in the face of COVID-19. Thailand’s UHC comprises the Universal Coverage Scheme (UCS), Civil Servant Medical Benefit Scheme, and Social Security Scheme, with the UCS financing access to nearly all healthcare services for previously uninsured populations [30]. Since the UCS is mainly funded by general tax revenues and it covers 70% of the population (mainly informal workers), while Taifacare in Kenya is mainly premium-funded through the SHIF [12,32], tax funding could be the reason that Thailand has surpassed Kenya in achieving UHC. Accordingly, the government of Kenya can prioritize increasing the tax-based funding of the SHIF in the medium term to replace premium contributions for people in the informal sector. Consequently, every Kenyan could access the same comprehensive care offered to public officers under the SHA’s enhanced medical schemes.

The likelihood of having health insurance was higher among literate and highly educated Kenyans aged 15–54 years compared to their counterparts with limited literacy and education. This pattern was also reported in a meta-analysis of 25 studies conducted in 12 LMICs, in which the most educated groups had 64% higher odds of having health insurance than the least educated groups [18]. In another study, the proportion of people without health insurance cover was reduced with an increase in the level of education, from 92% among Kenyans without an education to 77% among Kenyans with a tertiary education [21].

The odds of having health insurance coverage among individuals with higher education were nine times higher than the odds of having health insurance in a related study that analyzed the 2009 and 2014 KDHS data [13]. Active members of the NHIF were more likely to be highly educated and employed, while inactive members were mostly in the informal sector [6]. High education levels increase the chances of formal employment, and the contribution of formally employed people to the NHIF is compulsory. Hence, the attainment of UHC in Kenya through premium-based health insurance is dependent on Kenya’s progress in attaining high education levels and creating formal jobs, which are socioeconomic issues dependent on long-term government policy and political goodwill [33].

Health insurance uptake corresponds to the relative wealth levels of Kenyans aged 15–54 years, with individuals at the lowest wealth levels having a low likelihood of NHIF enrolment [34]. Lacking a TV, smartphone, and source of electric power, as noted in this study, corresponds to low wealth levels based on the KDHS 2022 [20]. The trend of poverty predicting health insurance coverage is consistent across all LMICs except Cambodia, whose wealthiest populations are less likely to be enrolled in the Colombian-subsidized regime, mainly because it exclusively targets vulnerable groups [18]. Therefore, the poverty-driven lack of health insurance in Kenya is more of a systemic political–economic issue than an individual-level issue, and it requires long-term, consistent poverty eradication interventions.

The unaffordability of premiums among Kenyans in the informal sector could be contributing to the inactivity of registered members [31], who have been further disincentivized by the NHIF’s inability to pay bills matching the incurred healthcare costs. Only a small fraction of Kenyans in the informal sector can afford the KSh 500 monthly premium, which is a sharp increase from the initial KSh 160 in an economy where the cost of living continually increases while low incomes persist [29]. Compliance with premium payments is likely to worsen in the informal sector with the SHA’s increased premiums for most people based on the means-testing system and the requirement for payment of the annual premium 14 days before the lapse of the annual period [12]. Allowing monthly contributions for SHA members in the informal sector would ease the burden of premium payment, consequently encouraging SHIF compliance and new SHA registrations.

High-earning people in the informal sector, who also mostly belong to social associations such as Matatu Savings and Credit Cooperatives (SACCOs), were more likely to be active members of NHIF compared to wage workers and self-employed small business operators [6]. The ability to pay the premiums of a health insurance scheme determines whether people will contribute to it [29]. Relying on the payment of premiums to achieve universal health coverage may be problematic in Kenya due to the large number of Kenyans living below the poverty line [13]. Thus, creating a supportive business environment that will increase earnings in the informal sector and enhancing social welfare programs for vulnerable individuals are necessary to increase health insurance coverage if Kenya is to rely on premium-based health insurance to achieve UHC [34].

Media factors such as having a TV influence the levels of health insurance coverage in Kenya, which similarly determine the demand for health insurance in other developing countries [35]. The NHIF mainly uses television and radio to communicate revised premiums and benefits packages to the public [31]. However, the revised benefits package after the 2015 NHIF reforms was seemingly inadequately communicated to the public [31]. Hence, the NHIF reforms may not have convinced non-registered Kenyans in the informal sector to be active NHIF members. Although 97% of Kenyans aged 15–54 years knew of the NHIF, possibly due to intermittently watching TV, several people in the informal sector lacked adequate levels of knowledge about health insurance schemes, perhaps due to the lack of a TV or smartphone. These media devices would have guaranteed unlimited access to NHIF information, thus increasing their probability of enlisting as active NHIF members [6]. People in the informal sector require better awareness of the services that they would access if they were contributing to the SHIF for evidence-based decision-making regarding their uptake of insurance cover [21]. Therefore, the SHA should create greater awareness through audiovisual channels such as TV and social media platforms to increase the uptake of health insurance in the informal sector.

Lacking a smartphone or a computer predicts low levels of health insurance coverage in Kenya. Digital access tools like mobile renewal programs influence the uptake of health insurance in Kenya, as in other LMICs [36]. Kenyans without smartphones are more likely to be uninsured compared to Kenyans with smartphones, since several Kenyans learn of health insurance through digital platforms [37]. The NHIF partly communicated its reforms through social media, website, and short message services (SMS), which require ownership of a mobile phone for access [31]. Therefore, the SHA should supplement digital media campaigns with in-person, TV, and radio campaigns to prevent the coverage inequities contributed by the digital divide due to the inequitable ownership of smartphones and computers.

The low likelihood of health insurance coverage among Kenyans aged 15–54 years without smartphones and computers could be due to limited access to health insurance-related information [36]. The digital customer interaction strategy at the NHIF relied on the ownership of smartphones or computers among Kenyans, and it influenced service delivery to beneficiaries as the digital public sought information about benefits packages and premium payments [38]. Moreover, considering the high rate of using MPesa to make payments, including premium remittances to the NHIF [39], the lack of a mobile phone may have impeded active membership in the NHIF. Addressing the digital divide in active SHIF membership requires supplementing the digital customer interaction strategy with a structured public outreach strategy in the short term to adequately inform people in the informal sector about the SHA.

Although media ownership may be a proxy for socioeconomic status [40], the inclusion of wealth levels in the multivariable logistic regression model that identified television, smartphone, and computer ownership as independent predictors of health insurance status ruled out the confounding effect of socioeconomic status. Digital infrastructure provides platforms for health literacy [41]; hence, they are likely to improve the understanding and consequently the uptake of health insurance [36]. The government of Kenya should implement strategies to increase the equitable ownership of digital devices in the medium term to avoid further deepening the associated disparities in health insurance coverage.

Age is a significant determinant of health insurance coverage status in Kenya, with Kenyans aged 19–25 years less likely to have health insurance compared to their counterparts aged 46–55 years. The NHIF covered secondary school students through a government-funded scheme called EduAfya between 2018 and 2022 [42], but it did not have a scheme for university students. Most 19–25 year olds are undergraduate students; hence, they are mostly neither employed nor under parental health insurance cover. Young adults in universities also have little knowledge about health insurance [2]; they only develop an interest in it as they age and transition into the workforce [43]. However, the Social Health Insurance Regulations No. 49 of 2024 addressed this challenge by providing health insurance for students up to the age of 25 years old [12]. Therefore, the proportion of 19–25 year olds with health insurance is expected to increase as soon as their parents become active SHIF members.

A recent need for outpatient care is also a significant predictor of health insurance coverage in Kenya. People who have recently paid for healthcare become active members of social health insurance, perhaps to avoid the burden of out-of-pocket care, especially after the NHIF reforms, when several outpatient services were covered [22]. Reimbursement for outpatient care reduces out-of-pocket expenditures for health and hospital admissions and improves patient outcomes [44]. Hence, it should be embraced by social health schemes such as the SHIF.

In addition, adverse selection, whereby patients’ healthcare is insured and individuals recovering from illnesses opt out of the insurance, is a significant challenge in the uptake of premium-based health insurance [45,46]. Therefore, awareness interventions to demonstrate the necessity of health insurance and diminish the perception of loss when active SHIF members do not need the covered health services should be implemented among Kenyans for continuous premium payments in both health and sickness [31]. The perception of loss can be further alleviated by strengthening the outpatient care package that most SHIF contributors need because only a few occasionally need inpatient care [44].

The main limitation of this study is that it used a cross-sectional study design; hence, causality could not be inferred since it was not possible to establish temporal relationships. However, the prediction of health insurance coverage status was reliably achieved from the identified associations by contextualizing the findings within the related literature. The KDHS data on health insurance only covered people aged 15–54 years and disproportionately included two-thirds of female participants; thus, they were not representative of the general population in Kenya. Nevertheless, the KDHS was the optimal approach to obtain quality data because of its geographical representativeness and complex survey dataset, which, once interpreted carefully, can inform national policy. Additionally, the survey did not include qualitative data that would have captured unmeasured confounders such as cultural beliefs; thus, insights that would have enhanced the understanding of the relationship between the identified predictors and health insurance coverage by addressing the residual confounding variables were unavailable, risking interpretation bias. The lack of post-SHA data and the inability to measure active premium contributions reduced the precision of the evidence in addressing the pertinent issues in the SHA. Nonetheless, the robust analytical approach used and the contextualization of the findings within the existing literature improve the real-world applicability of the findings.

## 5. Conclusions

The low uptake of NHIF coverage in Kenya persisted in 2022 based on the 2022 KDHS survey; it may have been among the motivations behind the government of Kenya’s transition to the SHA. The study identified low levels of education and illiteracy; poverty; the lack of a television, smartphone, or computer; young age; and recent outpatient care as the main predictors of low levels of NHIF coverage. The SHA should consider amplifying its media awareness campaigns and supplementing them with professional in-person campaigns to convince more people in the informal sector to become active SHIF members. It should consider enhancing its communication strategy to systematically increase SHIF awareness. It should also optimize the SHIF benefits package based on the available resources to make it more attractive to the informal sector through quality assurance in healthcare facilities, advancing progress towards equitable benefits for all Kenyans, refining the outpatient benefits package, enhancing the affordability of premiums, and exploring the option of allowing monthly premium payments. The national government of Kenya should consider accelerating the achievement of high rates of ownership of digital devices, the attainment of higher education and literacy, formal employment, and the establishment of formal businesses for equity in both contributions and benefits in national social health insurance. Most critically, Kenya should more intensively explore adopting a tax-funded health insurance scheme for all so as to optimize active SHA membership even among Kenyans in the informal sector. Future qualitative, cohort, and experimental studies should explore the effects of specific information dissemination interventions, particularly based on digital health and electronic health literacy strategies, on health insurance uptake in the informal sector in Kenya.

## Figures and Tables

**Table 1:** Demographic, socioeconomic, and health characteristics of the respondents covered by health insurance.

Characteristic	Respondents	Category	*n* (%)
Gender	14,232	Male	4844 (34)
		Female	9388 (66)
Age	14,232	15–18	557 (3.9)
		19–25	2937 (20.6)
		26–35	5292 (37.2)
		36–45	3974 (27.9)
		46–55	1472 (10.3)
Education level	14,232	Higher education	2811 (19.8)
		Secondary education	4402 (30.9)
		Primary education	5333 (37.5)
		No education	1686 (11.8)
Literacy	14,232	Read whole sentence	10,398 (73.1)
		Read only parts of sentence	1576 (11.1)
		Cannot read at all	2258 (15.9)
Wealth	14,232	Richest	2719 (19.1)
		Richer	3543 (24.9)
		Middle	2642 (18.6)
		Poorer	2284 (16.0)
		Poorest	3044 (21.4)
Has electricity	14,232	Yes	7814 (54.9)
		No	6418 (45.1)
Has a solar panel	14,232	Yes	4331 (30.4)
		No	9901 (69.6)
Has a radio	14,232	Yes	8868 (62.3)
		No	5364 (37.7)
Has a television	14,232	Yes	6618 (46.5)
		No	7614 (53.5)
Has a mobile phone	14,232	Yes	13,380 (94)
		No	852 (6)
Has a smartphone	12,360	Yes	6214 (49.7)
		No	6146 (50.3)
Uses internet	14,232	Yes	6747 (47.4)
		No	7485 (52.6)
Has a computer	14,232	Yes	1237 (8.7)
		No	12,995 (91.3)
Reads newspaper	14,232	At least once a week	1444 (10.1)
		Less than once a week	1853 (13.0)
		Not at all	10,935 (76.8)
Listens to radio	14,232	At least once a week	8734 (61.4)
		Less than once a week	1906 (13.4)
		Not at all	3592 (25.2)
Watches television	14,232	At least once a week	7017 (49.3)
		Less than once a week	1942 (13.6)
		Not at all	5273 (37.1)
Health status	14,232	Very good	11,229 (78.9)
		Moderate	2640 (18.5)
		Bad	363 (2.6)
Health insurance	14,232	Yes	4819 (33.9)
		No	9413 (66.1)
Insurance type	4819	NHIF	4478 (92.9)
		No NHIF	341 (7.1)
Received outpatient medical care in the last 4 weeks	14,232	Yes	3320 (23.3)
		No	10,881 (76.5)
		No data	31 (0.2)
Paid money for last outpatient care	3320	Yes	2879 (86.7)
		No	410 (12.3)
		Don’t know	31 (0.9)
Median outpatient cost	2879	Total outpatient cost	600 (IQR: 200–2000)
		Outpatient cost met in cash	500 (IQR: 150–1337)
Median inpatient cost	864	Total inpatient costs for overnight stay	8000 (3000–50,000)
		Inpatient costs paid in cash	4500 (IQR: 1000–10,000)

**Table 2:** Associations between characteristics of the sample and their health insurance status (1 = covered by health insurance), *n* = 14,232.

Patient Characteristic	Rao–Scott Chi-Square	Adjusted F	df1	df2	Significance
Educational level	1695.099	283.586	2.8	4458.4	0.0001
Literacy	547.487	164.689	1.9	2993.3	0.0001
Frequency of reading newspapers or magazines	341.212	80.643	2.0	3170.4	0.0001
Frequency of listening to radio	23.210	4.147	2.0	3124.9	0.0170
Frequency of watching television	809.344	188.858	2.0	3196.0	0.0001
Owns a mobile telephone	287.003	153.378	1.0	1599.0	0.0001
Owns a smartphone	782.297	335.464	1.0	1599.0	0.0001
Use of internet	1174.511	311.954	2.0	3159.1	0.0001
Frequency of using internet last month	1312.505	199.375	3.0	4714.4	0.0001
Self-reported health status	43.208	8.948	2.0	3171.5	0.0001
Has electricity	867.779	434.115	1.0	1599.0	0.0001
Has radio	247.649	95.860	1.0	1599.0	0.0001
Has television	1022.608	316.026	1.0	1599.0	0.0001
Has mobile telephone	184.067	106.876	1.0	1599.0	0.0001
Has a computer	695.256	265.554	1.0	1599.0	0.0001
Wealth index combined	1803.261	235.159	3.4	5449.2	0.0001
Household has a solar panel	81.416	47.775	1.0	1599.0	0.0001
Highest educational level attained	1816.833	285.006	2.8	4512.7	0.0001
Received outpatient medical care in last 4 weeks	13.109	6.679	1.0	1599.0	0.0100
Age (categorized)	68.890	8.807	4.0	6336.4	0.0001

**Table 3:** Results of the logistic regression model used to identify determinants of health insurance coverage (1 = covered by health insurance), *n* = 14,232.

Predictor	Category	B	Odds Ratio	95% Confidence Interval for Odds Ratio	*p*-Value
Highest educational level attained (Ref.: Higher)	No education	−1.595	0.203	0.153–0.270	<0.0001
	Primary	−1.298	0.273	0.227–0.329	<0.0001
	Secondary	−0.945	0.389	0.330–0.458	<0.0001
Wealth index (Ref.: Richest)	Poorest	−1.308	0.270	0.214–0.342	<0.0001
	Poorer	−0.863	0.422	0.346–0.514	<0.0001
	Middle	−0.590	0.554	0.473–0.649	<0.0001
	Richer	−0.499	0.607	0.535–0.688	<0.0001
Literacy (Ref.: No card with required language)	Cannot read	−0.622	0.552	0.409–0.746	<0.0001
	Partly reads	−1.29	0.879	0.750–1.031	0.186
Age group (Ref.: 46–55 years)	15–18	−0.592	0.553	(0.400–0.765)	<0.001
	19–25	−1.043	0.352	(0.298–0.417)	<0.001
	26–35	−0.529	0.589	(0.508–0.683)	<0.001
	36–45	−0.151	0.859	(0.740–0.999)	0.048
Has television (Ref.: Yes)	No	−0.487	0.614	(0.552–0.684)	<0.001
Has computer (Ref.: Yes)	No	−0.308	0.735	(0.624–0.865)	<0.001
Has smartphone (Ref.: Yes)	No	−0.222	0.801	(0.703–0.912)	0.001
Received outpatient care in last 4 weeks (Ref.: Yes)	No	−0.164	0.848	(0.769–0.936)	0.001
Solar panel ownership (Ref.: Yes)	No	−0.119	0.888	(0.799–0.987)	0.027

## Data Availability

The data analyzed in this study are deposited in Mendeley Data, a publicly accessible data repository. The DOI for the dataset is 10.17632/r4cy7xn5vk.1. The link to access the dataset is https://data.mendeley.com/datasets/r4cy7xn5vk/1 (accessed on 20 May 2025).

## Abbreviations

The following abbreviations are used in this manuscript:

KDHS	Kenya Demographic and Health Survey
NHIF	National Hospital Insurance Fund
SHA	Social Health Authority
SHIF	Social Health Insurance Fund
UHC	Universal Health Coverage
